# Embodying Transgender: An Analysis of Trans Women in Online Forums

**DOI:** 10.3390/ijerph17186571

**Published:** 2020-09-09

**Authors:** Pranee Liamputtong, Kyja Noack-Lundberg, Tinashe Dune, Brahmaputra Marjadi, Virginia Schmied, Jane Ussher, Janette Perz, Alexandra Hawkey, Jessica Sekar, Eloise Brook

**Affiliations:** 1 School of Health Sciences, Western Sydney University, Campbelltown, NSW 2560, Australia; p.liamputtong@westernsydney.edu.au; 2 Translational Health Research Institute, Western Sydney University, Campbelltown, NSW 2560, Australia; KNoackLundberg@lincoln.ac.uk (K.N.-L.); b.marjadi@westernsydney.edu.au (B.M.); v.schmied@westernsydney.edu.au (V.S.); j.ussher@westernsydney.edu.au (J.U.); j.perz@westernsydney.edu.au (J.P.); a.hawkey@westernsydney.edu.au (A.H.); j.sekar@westernsydney.edu.au (J.S.); 3 School of Medicine, Western Sydney University, Campbelltown, NSW 2560, Australia; 4 School of Nursing and Midwifery, Western Sydney University, Parramatta, NSW 2150, Australia; 5 Gender Centre, Annandale, Sydney, NSW 2038, Australia; e.brook@gmail.com

**Keywords:** trans women, transgender identity, online forums, unobtrusive method, gender dysphoria, gatekeeping

## Abstract

This paper discusses the way that trans women embody their transgender identity, focusing on identity questioning, gender dysphoria, clinical gatekeeping and medicalized narratives. Situated within the hermeneutics methodological approach, we adopted the unobtrusive research as our research method, where data was derived from online forums where trans women posted content about their perspectives and experiences of gender and gender transitioning. Thematic analysis method was used for data analysis. Our findings suggest that gender identity is embodied and socially negotiated. Many trans women were initially ambivalent about their transgender identity and some continued to question their desired identity throughout adulthood. When presenting to healthcare professionals many trans women reported being expected to adopt a ‘wrong body’ narrative in order to gain access to treatment and surgery for gender transitioning and affirmation. In doing so, trans women interact with significant others and health care providers, and face many challenges. These challenges must be understood so that trans women can perform self-determination practices as a way to achieve gender autonomy.

## 1. Introduction

The term transgender (trans) refers to individuals whose gender presentation differs from their sex assigned at birth. The term transgender includes diverse identities such as trans women, trans men, non-binary, gender queer, fa’afafine, brotherboy, sistergirl, two-spirited, gender fluid or gender-nonconforming [1,2]. Frameworks to understand transgender identity are often premised on antiquated theories based on gay and lesbian identity development [1]. Such theories propose that gay and lesbian people may feel initial anxiety or confusion and choose to remain “closeted”, before progressing to develop an actualized and fulfilled identity, where anxiety is replaced by pride [3,4]. Transgender identity development is not linear and may fluctuate over time. Research with trans women has shown that gender transitioning and affirmation is generally a positive experience associated with improvements in mental health, including reduced symptoms of depression, anxiety, and suicidal ideation [5,6]. However, gender transitioning can also result in challenges, such as family rejection, risk of employment discrimination and violence [7,8,9]. Religious advisors and mental health professionals who oppose the validity of the trans experience, even today, pressure trans people to surrender their deeply known identity despite the fact that attempts to change gender identity or prohibit them from being transgender are harmful, ineffective, and abusive [10]. As a direct result of these pressures, a small minority of trans people do de-transition, albeit often temporarily [10,11]

Since the publication of an authoritative monograph ‘*The Transsexual Phenomenon*’ by sexologist Harry Benjamin, trans women have been expected to present according to guidelines which detail what constitutes a real trans woman [12]. These guidelines were premised on one practitioner’s account of working with what Benjamin describes as “transsexual” women. In order to distinguish ‘real’ trans women from those who may be experiencing mental illness, or who may not actually be transgender, trans women’s experiences have been expected to follow clear narratives. These narratives include a clear knowledge of being born in the wrong body, a lack of interest in typical gendered childhood pursuits, and strong body dysphoria focused on the genital area [12]. Sandy Stone’s seminal text, ‘The Empire Strikes Back: A Post-Transsexual Manifesto’ details how clients’ gender presentations so accurately mimicked the presentation detailed in Benjamin’s work [13]. This presentation impressed practitioners, until they realized that clients had been studying the book in order to be granted access to medical care. The key criteria for acceptance for treatment was a feeling of being “in the wrong body” [13].

Today, there are multiple models aimed at improving the health of transgender people, including the World Professional Association for Transgender Health (WPATH) Standards of Care version 7 [14] and informed consent/shared responsibility models. Diagnostic models adopted in clinical practice require a diagnosis of gender dysphoria (a feeling of persistent discomfort with assigned gender or biologic sex) according to the criteria in the current Diagnostic and Statistical Manual of Mental Disorder (DSM-5) prior to treatment for gender transitioning [15]. Clinical guidelines stress the importance of establishing a clear diagnosis through differential diagnosis and thereby avoiding harm to the patient. Conversely, informed consent models stress patient self-determination and collaboration with healthcare models in order to get healthcare needs met [16,17]. For instance, under the standards of care model, a psychologist or psychiatrist referral is made, usually involving 3-6 assessment sessions in order to make an official diagnosis of gender dysphoria. According to the Australian Healthcare Associates [17], the WPATH Standards of Care version 7 specifies that to access hormone therapy, there is no requirement for trans people to undergo a psychiatric diagnosis. However, trans people still need to obtain a referral letter from a mental health or psychosocial assessment by a mental health clinician or other experienced health professional for them to have access to hormone therapy. Additionally, to access gender affirming surgeries, a psychiatric diagnosis is still needed.

In contrast, within the informed consent model, a diagnosis is not seen as necessary for access to treatments for transitioning, such as hormones [12], and there is evidence that general practitioners (GPs) are able to support trans women based on their experience in providing hormone therapy to cisgender women [18]. Referral to a psychologist or psychiatrist is only made for the patient to discuss potential consequences of social and medical gender transitioning [15]. Patients may also be referred to endocrinologists, where GPs have inadequate training in transgender health. 

Currently, trans women living in different Australian jurisdictions may receive very different medical treatment associated with gender transitioning. Few clinics adopt an informed consent model, and those that do are most often private clinics and practitioners. Public clinics rarely offer specialist support to trans women for gender transitioning [19]. Due to perpetual discrimination and limited educational or employment opportunities, trans women are overrepresented in lower income brackets [2,20]. This presents a significant obstacle for trans women in accessing treatment at private healthcare facilities. While some trans women found obtaining treatment and support relatively straightforward, others report receiving treatment that was invalidating of their gender identities, stigmatizing, or in some cases abusive. Financial concerns were often seen as a barrier to accessing medical support to transition [2,20]. While transitioning is sometimes positioned as a ‘want’, lack of support can result in heightened gender dysphoria, severe distress and potentially self-harm or risky coping strategies such as drug and alcohol use for some women [21].

In the past year in New South Wales (NSW), Australia, there have been changes after a prominent endocrinologist retired, leaving many trans people in limbo without access to Gender Affirming Hormone Therapy (GAHT). In response, ACON (2020) has created TransHub and helped find doctors including GPs and endocrinologists who will provide GAHT for trans people to fill this need. There are more practitioners in NSW now offering GAHT and some of them work under the informed consent model. According to ACON’s TransHub, at least 27 practitioners/clinicians practice under the informed consent model in NSW (see https://www.transhub.org.au/doctors). However, access to gender affirming healthcare in NSW, and across Australia, is still in crisis. Despite the addition of several GPs, it is still very difficult for many trans people to access these medically necessary interventions in a timely manner, where they live, that is safe and self-determined, and also avoids medical gatekeeping. (Teddy Cook, personal communication, 7 August 2020) [22].

In this paper, we discuss the way that trans women embody their transgender identity, focusing on identity questioning, gender dysphoria, and clinical gatekeeping and medicalization narratives. Our data is derived from online forums where trans women have posted content about their gender perspectives and experiences of gender and gender transitioning. This study is based on a larger, mixed-methods research project that explores gender transitioning and sexual violence towards trans women from culturally and linguistically diverse backgrounds [9]. This paper contributes to the discussions regarding the embodiment of transgender identity amongst trans women in the health social sciences field.

### 1.1. Embodying Transgender: Theoretical Framework

Theoretically, it is argued that gender is constructed through social interaction. Accordingly, our gender is recognized in our everyday interaction. Social construction theory suggests that individuals learn about their gender role through socialization (social interaction) within the family and society from a young age [23,24,25,26]. Transgender individuals circumvent traditional models of gender, challenge the “*normative coercion to perform gender dichotomously*” [27]. For many transgender individuals, their transgender identity is experienced as both innate and socially negotiated [1]. Polderman et al., in their review of gender identity, argue that gender identity and its related socially defined gender constructs are partly impacted by biological elements [28]. In their research on butch and femme lesbian genders, Levitt, Gerrish and Hiestand [29] and Levitt and Hiestand [30] suggest that gender was experienced as biological or deep-seated as well as being socially constructed. This phenomenon has been demonstrated in Ussher et al.’s study [9] with trans women of color in Australia. As traditional gender norms do not occur for individuals outside the gender binary, transgender individuals try to find not only “*an answer as to who they are, but also an answer as to what ‘who I am’ even means*” [8]. They are constantly questioned about their gender identity by family and others. Paradoxically, they find that they have to discover their own gender identity, often with confusion, self-doubt, and immense anxiety [31].

Not only do transgender persons themselves question their own identity, they are also questioned by health care providers when they seek health care. The process of transitioning is a common discourse amongst transgender individuals [8,32]. Transitioning connotes “*the overall process of aligning one’s gender expression with that of their gender identity*” and to “*renounce the gender* [that was once] *ascribed as a result of their birth sex and to adopt the gender with which one identifies*” [8]. In this sense, transitioning is not a “*singular event*” but “*a continual experience*” which may eventuate over several years [31,33]. Some transgender individuals undergo surgeries and hormone therapy to affirm their identified and desired gender; others may choose not to pursue them [2,8,32,33]. For those who opt for surgeries, this choice requires them to interact with health care providers, which can add another layer of confusion, self-doubt and great stress to their lives. As we shall show, some trans women have to deal with uneasy interactions with their health care providers when seeking health care.

### 1.2. Online Forum and Gender Space

As the boundary between the virtual (or online) and face-to-face communication interactions blurs, identity formation is more influenced by and takes place in online spaces. This online space is more prominent for marginalized groups who may not be able to express and develop their identities in public or at home [8,34,35]. Young people, in particular, often search for information about their gender identities online [36,37], even if they are not aware of terms such as transgender or genderqueer. An overwhelming 98% of Smith et al.’s research [37], participants named websites as an important source of information on gender, while only 57% and 55% of participants placed importance on support groups and health professionals, respectively. People may join forums or support groups to discuss their identities, and their identities are often shaped discursively through discussions with others. This includes debates and consensus around identity group boundaries and identity formation processes [38,39]. For example, in Cavalcante’s study [38], his twenty-one year old interview participant asserts: “*I did it all online*”, referring to her exploration of gender identity and transition. Cavalcante [38] argues that recent media and technology developments have facilitated movements towards more mediated transition. He characterizes online forums as “care structures” or “*architectures of organized care and concern*” [38], where connections are formed and a sense of belonging is created.

Trans people use online support groups to form friendships and connections with other transgender individuals [39,40]. Because transgender people are a minority and only make up a small percentage of the population, many people may have had limited contact with transgender communities, due to stigma, isolation or anxiety, and because transgender people are a minority and only make up a small percentage of the population [36,39]. According to an Australian research project [37], more transgender and gender diverse people were involved in online forums (62%) and online support groups (48%) than in face-to-face support groups (40%). A substantial proportion of gender diverse and transgender individuals felt more content in their identity after being involved in online forums (37%) and online support groups (36%).

## 2. Materials and Methods

In this paper, we utilized the unobtrusive method that involve data collection where the collection does not influence the outcomes, as there is no direct interaction with participants [41]. Such methods include analyzes of published materials, archival research, and online forums, among other methods. These methods produce more ‘naturalistic’ data. Due to anonymity, participants are likely to be less restrained in discussing sensitive or taboo topics [34,35]. Trans women who are willing to discuss their experiences of sexual violence are a small and hard-to-reach group [34,35], and online forums can provide access to rich data on this topic. The unobtrusive method is situated within a hermeneutics methodological approach. Hermeneutics offers a theoretical standpoint that researchers can use for their interpretive understanding of the data (in the case of this paper, it is the text posted online by trans women) with special attention to context and purpose [35]. Data collection within hermeneutics has its foundation in the act of writing [35]. Such data include lived experiences written in diaries, journals, and posted on online forums. Hermeneutics is employed extensively in the unobtrusive methods where researchers attempt to understand written texts from published materials [35].

Online support groups on Facebook were contacted, however none responded for requests to participate in the research. Since many online forums or subforums specified ‘no researchers’ (meaning the use of data for research purposes are prohibited), the project was limited to forums that did not make this stipulation. We also excluded online forums that lacked recent engagement (the forums were inactive for a while). The forums chosen were the subreddits r/asktransgender, r/transsupport, and r/transgenderau, along with one relevant thread from TransPulse Forums. Other online forums for trans women were excluded due to lack of recent engagement. Threads were selected based on relevance to themes, length of thread, and recency. The selected threads were imported directly from the websites using NVivo’s NCapture (QSR International, Melbourne, Australia) tool.

Thirty-nine threads were chosen that were focused around the project’s research themes of violence and safety, being trans and a person of colour, and trans identity and health. As we selected for more recent threads, over half (20) of the threads were posted within the past few years (since 2017) and all except eight were posted less than five years ago (since 2015). The oldest thread was posted in 2012. In total thirty-eight original posts, and 950 comments were coded using NVivo 11 (QSR International, Melbourne, Australia). The longest thread contained 103 comments in response to the original post, and the shortest contained five comments in response to the original post.

Thematic analysis method was adopted in order to construct patterns and themes in the data [42]. Codes were generated iteratively through multiple readings of the text. Codes were then examined for patterns, and these identified patterns were reviewed to construct themes. The second author did the first round of data analysis and cross-checked by the first author. The generated codes and constructed themes were discussed between the first and second authors to ensure a credibility of the constructed findings. The findings were also checked for credibility by a trans woman. Thick descriptions of our findings are provided in the Finding section. Theory is used to assist in the interpretations of our data. Safety, identity, transitioning, discrimination and dysphoria were the key themes that identified. This paper focuses on the key themes of identity, transitioning, and dysphoria, while another paper is concerned with safety and discrimination [39].

As this study analyzed data from online forums which are a public space, we did not require an ethic clearance from our institution. However, we ensured that there is no true identity of any forum posters can be revealed in writing up this paper.

## 3. Results

Three key themes were generated from the online forum: Questioning Identity, Gender Dysphoria (GD) and Ambiguity, and Gatekeeping and Medicalized Narratives of Transgender People: Blocking or Opening the Way. These themes will be discussed below. It should be noted that in presenting our findings, we incorporated literature and theory to support our interpretations of the findings.

### 3.1. Questioning Identity: ‘HELP I Have No Idea What I Am’

Transgender identity is often framed in popular culture and medical discourses as being ‘born in the wrong body’ or always knowing ‘that they were in some way different from everyone else’ [12]. Some trans women, following these narratives, provide evidence of how they always liked dressing up as a girl, for as long as they could remember. However, while some trans women ‘always knew’, others do not follow such a clear path. Many threads in the online forums focused on being unsure about one’s transgender identity. Some forum posters whose experiences fell outside this clear narrative felt uncertain or worried about whether they were ‘really’ trans. The forum poster below, for example, “never had like a ‘moment’ or anything of realization”. This quote indicates that some people expect a clear knowledge of one’s gender identity to emerge, either in a moment, or through a linear process. However, many participants experienced vacillation and non-linear processes of ‘uncovering’.

*When I was a kid I would steal my sisters clothes constantly and wear them in private. Now that I’m an adult I have my own stash of clothes and makeup that I use. I also constantly think about transitioning and am envious of women and other MtFs [male-to-female trans people] that pass really well. However I never had like a ‘moment’ or anything of realization. I would prefer the body of a woman, but I dont feel “wrong” or anything presenting as a man. This kinda freaks me out because it makes me feel like I might not actually be trans, and if I were to transition I might regret it.*

However, some other forum posters emphasized coming out as a “process” of exploration, rather than people needing to make a clear decision or choice. Because transitioning was a process, people could explore gender identity as much or as little as they wanted before clearly deciding to come out or transition.

*You don’t have to get everything settled and sorted and go from nothing to “OMG DEFINITELY I AM TOTALLY A WOMAN AND NEED TO DO EVERYTHING RIGHT NOW”—its a process. You can come out as questioning or exploring your gender, you can come out as “probably trans”, you can come out as trans and then you can say “oh well didn’t work so well so I’ll try something else.”*

Some forum participants were unsure, or had not initially been sure if they were trans, but were envious of trans women who were able to express femininity, or wanted to be a part of the trans community.

*All those transgender people who are transitioning are sooooooooooo lucky. But I’m not trans.*

*There, I said it—I wish I was actually trans, I wish I hated my male body so I could be an actual part of the trans community, I wish I related to all the trans people who have dysphoria every day. I wish I was able to feel a real connection to femininity instead of just feeling like absolute nothingness inside. It’s been months now and I’m still no closer to actually transitioning because I have to be 100% sure before I actually do—if I come out to my parents, friends, the whole world, and then realize I’m actually not trans, then I think I might just off myself. That fear of being the stereotype of a fake trans person, a “transtrender,” someone who’s just doing it for attention, has been my fixation for so long—I wish I could just go back to being vaguely unhappy but oblivious about gender stuff altogether. It feels like whatever I do, whether I decide to transition or not, I’m going to go to my grave regretting it—if I do transition falsely, that makes me a failure and a traitor, but if I don’t and it turns out I should’ve, that’s basically the nightmare scenario, like putting the right answer down on a multiple-choice test but then crossing it out and putting the wrong one… except instead of meaningless points, it’s my entire life and happiness on the line.*

The term ‘transtrender’ signals someone who is not really trans, or is only adopting a trans identity due to it being seen as fashionable [43,44]. This term appeared frequently on the forums. The fear of being a ‘transtrender’ or being seen as a transtrender by the community was quite common, and attested to the stigma carried by this term. This stigma potentially increased the distress of those who were going through the questioning process, as they did not experience the more commonly-endorsed clear and certain narrative, to a moment of realization, and they were concerned about not being accepted by the community. ‘Transtrender’ seems to function as a boundary-marking device, identifying what kinds of people count as authentic or real trans people, and excluding others who do not meet their criteria [44].

### 3.2. Gender Dysphoria (GD) and Ambiguity

Related to gender questioning, gender dysphoria (GD) was a term that was disputed among forum participants in this study. GD refers to a feeling of persistent discomfort with assigned gender or biologic sex [45,46]. Experiencing GD and receiving a diagnosis was key to being able to medically transition [47]. Because, for most, the diagnosis of GD enables transition and ‘confirmed’ transgender status, the definition is heavily contested [47]. Current International Classification of Diseases (ICD) guidelines stipulate that for GD diagnosis someone must experience distress or impaired functioning due to distress related to the dysphoria [48]. As GD is strongly associated with genital-anatomical dysphoria [49], many forum participants were uncertain about whether what they felt was GD. Others said they were unsure whether they had felt GD at all.

*I’m not sure I’ve ever met a trans person who had dysphoria every day. For many of us, myself included, it comes in wave. We’ll experience intense dysphoria at some moments, but no dysphoria at others. I can go months at a time without being dysphoric, but that doesn’t mean it’s any less real when I do feel that way.*

GD was experienced differently by forum participants. Some participants described it as a feeling of ‘nothingness’, rather than bodily hatred or an attraction to femininity.

*You already have a way out of all this. You can transition. Angst about being labeled a “transtrender” aside, everything you are describing that you experience sounds exactly like gender dysphoria. I didn’t experience dysphoria as a hatred of my male body. And pre transition I didn’t feel a real connection to femininity. Instead, I felt like absolutely nothing inside. Nothing made me happy except escaping from reality. I longed to somehow be female but I somehow never connected this longing to “being trans;” nor did I ever consider that my other feelings could mean I was trans. But in fact, the longing, the envy of women and their lives, the feeling of nothingness, of perpetual emptiness, the dissociation from my body, the constant desire to escape myself, all of these were gender dysphoria.*

Some forum participants felt GD more strongly at some times than others.

*Gender dysphoria doesn’t hit all at once like a ton of bricks. It creeps in, often starting around puberty and growing during your teen years. Some people knew they were transgirls at age 8. Some of us put on a skirt at 16 and were like, “this feels… right.” Just throwing it out there, the people who knew at age 8 tend to be louder and prouder, so you hear their stories more but trust me, lots of us were way more confused than that.*

Another forum participant described feelings of ‘gender euphoria’ instead, which helped to provide a feeling of confirmation of gender identity.

*Not all trans people experience dysphoria. Some experience the opposite, gender euphoria when affirming their neurogender (gender identity). Some experience glee when they try their first dress on, and some get all warm and fuzzy inside when they look down and see cleavage. It doesn’t even have to be sterotypically male or female since there is no right way to be either a woman or a man.*

Trans women’s lived experiences of gender dysphoria (GD) and gender incongruence (where there is discrepancy between gender identity and physical sex characteristics) contrast with common medical narratives of gender [12,47]. The lived experiences present a picture of great complexity, where narratives of ‘being in the wrong body’ [10,48] and strong genital-anatomical dysphoria co-exist with other narratives of euphoria and a sense of affirmation at finally feeling a sense of gender congruence, numbness or emptiness, and intermittent, rather than constant dysphoria. While guidelines, by necessity, simplify or reduce experiences, further exploration could be undertaken into the broad range of manifestations of GD and gender incongruence. With other stigmatized identities, including those in the LGBI community, it is recognized that an individual might experience any of a wide range of reactions to not being congruent with the broader community, including joy, relief or pride [47]. Such an awareness need not necessarily manifest in embodied distress, as required by the ICD Guidelines [48], yet it is no less valid.

### 3.3. Gatekeeping and Medicalized Narratives of Transgender People: Blocking or Opening the Way

As previously mentioned, there is a long history of trans people forming and rehearsing narratives to better meet the requirements of ‘gatekeeping’ health professionals [50,51]. In most countries trans people need psychiatrists to sign off on their paperwork to confirm that they meet the requirements for GD diagnosis in order to transition [33,51]. These requirements render distress in many trans individuals [52,53]. Our data indicate that some health care practitioners impose an understanding of gender identity that goes beyond the medical requirements. Forum participants acknowledged there were more practitioners who were knowledgeable about trans experience due to the increase in referrals and transgender identification. However, some forum participants still encountered practitioners who lacked an understanding of gender identity, were deemed unprofessional, or behaved in an openly hostile manner. Due to negative encounters, or hearing of others’ distressing encounters, many posts expressed distrust, fear and hostility toward psychiatrists and psychologists in particular, as well as GPs, as reported in other studies [47,51]. The forum participant below talked to a GP who seemed to conflate gender and sexuality, focusing on her sexual attraction to men, and her mental health issues. The heavily discredited idea that many people regret transitioning was also raised.

*I had my appointment with a GP to get a referral which I did end up getting but, I felt as though the GP really didn’t want to help. Firstly she asked about my family and I mentioned I was the youngest in a line of several brothers and that my father had left when I was young, she asked how my experience was growing up, then she fixated on my sexual attraction to men “do you feel you are attracted to men because they have something you wish you had?”, I JUST TOLD YOU I DON’T WANT TO BE A MAN, so what not only do I just have gender dysphoria because daddy left, I have an attraction to men because I’m jealous of them? Then she insisted I understand that gender transition is not a cure for anxiety and depression and a lot of people regret transitioning and I’m thinking “no shit woman, I’ve topd you i’ve mostly overcome my depression”. Uggh I just, I’ll deal with this soon.*

Another forum participant’s GD was questioned by a psychiatrist as she did not fit the narrative of ‘always knowing’ she was trans and showing signs from a very early age. This led the forum participant to ‘go back in the closet’ about her gender identity and become reticent to see psychiatrists in future.

*The very first time I opened up to a psychiatrist about my gender dysphoria he laughed at me. He said I could not be transgender because I didn’t show signs in early childhood. I was 15 years old and it still sticks with me. It was one of the things that made me go back in the closet and try to convince myself that I definitely couldn’t be trans. I fear the next time I have to open up to a psychiatrist about this. I’ve not had good experiences with them.*

Another participant faced a situation where the therapist was abusive and screamed at her that she would “make a fucked up girl”.

*I was trying to see a therapist about my gender dysphoria and at one point he screamed at me “[deadname] you’re a fucked up boy and you’ll just make a fucked up girl.” I had to really assert myself, had to demand that I was trans, to everyone, before people would accept I was trans.*

*I checked myself in the mental pokey after about eight months of transition or die. I told them all my symptoms and they said “You’re not trans, you’re a borderline personality”… essentially saying I was hysterical.*

Other participants faced more everyday forms of ill treatment, which, while less severe, impacted on how comfortable they felt. Misgendering was raised as an issue. A forum participant attending a consultation to support a friend was misgendered and assumed not to be trans. Other practitioners were seen as having a lack of understanding of trans issues, a phenomenon found even among practitioners who were billed as ‘trans friendly’ or were on lists of providers with an understanding of trans health [47].

Forum participants were aware that doctors and psychiatrists had the power to restrict or deny access to transition, including hormones and surgery [47]. Many felt that this power was an imposition, as only an individual can ‘know’ what their gender identity is. One participant drew a parallel with sexuality, stating that it was not truly possible to diagnose whether someone is gay or not, so people should be able to make a choice about whether to transition or not. The participant below was aware of the way psychiatry has shaped narratives around what it means to be trans.

*Psychiatrists and psychologists have a long history of treating us like garbage. There are only a few enlightened individuals in a sea of misunderstanding currently. This is because of the legacy of gatekeeping and the idea that all trans people have to fit into a very specific narrative or that we must feel intense distress. The reality is that you can feel incongruent (have dysphoria) without that severe distress, and most of us do not fit into any particular narrative. This does not make you less trans.*

It was clear that trans women were still aware of the potential expectations of some practitioners that they will follow certain narratives, and are adapting their narratives to make sure that they meet the DSM-5 criteria, regardless of whether their experiences of being a trans woman are in line with these definitions. The forum participant below (from the United States) was concerned that she could be denied access to services:

*I was so terrified of gatekeeping (deep South) I studied the DSM5 criteria so I’d be able to hit all the notes.*

For this participant, their therapist turned out to be accepting, although the participant was cognizant of common attitudes and had prepared well in order to avoid being turned away from accessing services.

Some forum participants would often suggest distressed trans persons seek help from gender therapists, which demonstrated more trust in therapists with specific training in supporting people with gender identity issues than in medical professionals. However, not all gender therapists met everybody’s needs. For example, a gender support service was seen by a participant to be more focused on its clients following a clear teleological transition narrative, with an endpoint as having successfully completed sex reassignment surgery. While the participant did not express purely negative feelings toward the service, and would not dissuade people from going, she felt the service’s approach did not cater to the broad range of transgender identities and needs.

*I really wouldn’t want to put anyone off going there! My feeling is however that the regard the destiny of every trans person is to transition fully. This is fine for many, but for those who aren’t so keen on that narrow commitment (such as for example not having surgery) it’s a little…exclusionary?*

## 4. Discussion

In this paper, we discuss the embodiment of transgender identity of trans women using data from online forums, focusing on identity questioning, gender dysphoria, clinical gatekeeping and medicalized narratives. Among forum posters, transgender identity was complex, with many forum participants questioning their transgender identity for long periods, before achieving certainty, as others have reported [8,32]. This finding contrasts with commonly-accepted narratives of transgender women ‘knowing’ from an early age, or at least from childhood that they did not fit with traditional gender roles, or felt like they were in the wrong body [12,50]. Further, gender dysphoria was conceptualized and experienced as more complex than the more commonly understood genital-anatomical dysphoria. The narrative of trans woman knowing that they were always trans was true for some trans women but not for others. Some trans woman had a clear sense of gender identity from a young age while others did not. Some trans people experience genital gender dysphoria while others experience dysphoria based on their gender expression, identity or from the way they are treated by society. Some forum participants in our study did not experience high levels of distress, while still maintaining transgender identities and strong desires to transition socially and/or medically.

In the past decade, there are far more young people exploring their gender identity due to the increase in information and representation of trans people online and across other media [54]. While awareness of transgender identities has increased dramatically in recent decades, forum participants still reported negative experiences of receiving invalidating or hostile treatment. Many forum participants felt that healthcare practitioners such as GPs, psychiatrists and psychologists were ‘gatekeeping’, opening or blocking them from being able to medically transition by declaring their experiences of gender incongruence as invalid, and not facilitating access to hormones or surgery, or referrals to specialists who may be able to provide these treatments [47,51].

Our findings suggest that gender identity is embodied and socially negotiated [8,24,33,55]. Many forum participants were initially ambivalent about their transgender identity and some questioned their desired identity. They are engaging in the sense-making of their embodied and negotiated identity [24,55], which signifies that gender transitioning can be a more fluid process than previously identified [2,8,33]. One’s internalized sense of gender is an intrinsic instinct for trans women; it is an instinct to try on girl clothes, or find themselves associate with things they are not supposed to in a transphobic society. They may not be clear-cut but it is present. Many trans people have confusing journeys but as they overcome their confusion, repression and conditioning, they discover a strong identification with their particular gender identity. The process is fluid and the ‘wrong body’ narrative does not apply to everyone, but one’s internalized sense of gender is an automatic instinct the moment they feel that they are different even if that gender conception is not fully formed [50].

Our analysis of forum posts demonstrates that many trans women are still expected to present in accordance with a clear ‘wrong body’ narrative in order to gain access to treatment and surgery [12,50]. Some forum posters also described formulating a clear gender narrative based on perceived clinical expectations in order to receive treatment. While from the clinicians’ point of view such narratives can erase ambiguity of experience and presentation, and reward gender identities that fit clearly within a binary two-sex model, the narratives may not be true for the lived experiences of the trans persons, and thus negating or invalidating those experiences. The clinical encounter, then, become a staged process rather than a true patient-centered care [56]. Further, the notion of requiring a diagnosis and treatment is particularly difficult for non-binary or genderqueer people, who may wish to change their bodies to fit with their internalized sense of gender [57]. However, their sense of gender may not be congruent with social ideals of gender, and therefore a diagnostic model, with treatment aimed to achieve standard gender congruence, would not be appropriate [33].

Gender dysphoria (GD) in the classical, genital anatomical sense, including major distress, was not experienced by all forum participants and fluctuated over time. This finding was similar to others’ in which GD was contextual [46]. For example, in Osborne and Lawrence’s study in a jail [46], GD was often exacerbated in that context as participants were not always able to express their gender identity in the same way as they could outside the prison context, due to issues such as access to hormones, cosmetics and clothes through which they could express their identities.

The medical model often presents GD as originating within the individual, due to ‘being in the wrong body’ or having an intrapsychic sex incompatible with one’s sex characteristics [12,50]. Health practitioners pathologize gender dysphoria as a psychological disorder simply because a person of a connoted gender exhibits traits of another gender has a desire to live as their true gender. This view belittles the multiple means through which “*gendered lives are … lived*” [33]. However, diagnosis remains necessary for many trans people who wish to pursue a legal change in status or undergo gender affirmation surgery or treatment.

Research has also revealed that it is often the social stigma of being transgender and experiencing everyday difficulties such as not passing when performing everyday activities that contribute to dysphoria and distress [8,20,53,58]. This finding draws on the minority stress framework [59,60] in which many members of minority groups including sexual minorities, face stress and a higher prevalence of mental health issues, not due to internal pathology, but due to facing prejudice on a daily basis [8,47,58,61,62]. Dysphoria may be heightened not only due to an internal awareness of non-congruence, but an ongoing awareness of non-congruence with socially-sanctioned gender presentation, and the everyday violence such as stares and comments that one may face in that situation [22,47].

Medical practitioners adopting a diagnostic model may be following a paradigm where the emphasis is on harm avoidance, differential diagnosis, and ensuring any co-morbidities that complicate treatment, such as mental illnesses or conditions that might interact negatively with hormones, are addressed. When being transgender is conceptualized as a diagnosis under older rubrics such as gender identity disorder (GID), or the more recent gender dysphoria [46], the existence of a diagnosis facilitates the assumption that there is an issue that can be treated [33]. The move from gender identity disorder locates the issue from being situated within the identity, to the dysphoria or level of distress experienced when one’s body does not conform with one’s gender identity [33].

Accounts of experiences with health services presented similar findings to previous studies undertaken in Australia, the United Kingdom, and the United States [47,58,63,64]. In Australia, participants in Hyde et al.’s study on transgender mental health [65] “*overwhelmingly*” felt that “*their health needs were not being met*”. There were also concerns about discrimination and healthcare providers’ lack of knowledge about transgender health [64,66]. A study in the UK also found participants experiencing verbal abuse, misgendering and disrespect by practitioners [63]. Many survey respondents in Ellis et al.’s study believed that mental health practitioners needed more education on transgender health, and that they had limited awareness. In another study [33], some practitioners doubted the participants’ gender identities which did not conform to accepted gender norms, causing the participants to sense that they were treated as the ‘Other’.

While the World Professional Association for Transgender Health [14] has issued a clear set of clinical guidelines for practicing transgender health, many forum participants experienced being treated in ways that contradict these guidelines or evidence outdated modes of conceptualizing transgender identity. For those who desire gender affirmation surgery, medical providers, particularly doctors, act as gate keepers who can ‘block’ or ‘open’ access to such treatments [51]. Transgender individuals come under their scrutiny and therefore may become “subject to repressive ideas” held by some medical practitioners. There are medical practitioners who believe that all transgender people who qualify for sex reassignment surgery must be “‘*heterosexual’ (oriented sexually toward the gender other than the one they claim)*” [51]. Whitehead and Thomas [67] have also revealed that many medical professionals will only provide sex reassignment surgery if they perceive the identity to be the transgender person’s ‘true’ core gender identity. This subjective external view of a ‘true’ transgender identity is also evident in psychiatry [64,68,69]. Thus, it is essential that extensive consultations between trans women and health care providers occurs. This can avoid misunderstanding and mismanagement of trans women who wanted to engage in gender transitioning. Clinicians need to understand the dynamic nature of each trans woman’s journey and work with them to ensure appropriate clinical decisions are made at the right time. When there is a discrepancy between medical considerations and the wish of the trans woman, the clinician needs to explain their decision empathetically, preserving patient-centeredness [56]. Additionally, improvements in medical services for transgender people, particularly around their transitioning journey, would require improvements in inclusive, non-binary gender teaching at medical schools as well as in continuing professional development, as part of a broader improvement in gender teaching in medicine [70,71].

We acknowledge that there are some limitations of this study. As the analysis was based on online forum analysis, it was not possible to ask any follow-up questions for clarification or further insight, as one might in a semi-structured or unstructured interview. However, this is a limitation that is also held by short answer questions in questionnaires, where the comments are analyzed and coded without the ability to follow-up or clarify. The amount of demographic data that was available for collection was limited to self-identity labels, where provided, therefore location, age and ethnic background were not always clear. The closed nature of many online transgender social media groups, combined with the fact that several members of our research team do not identify as transgender, meant that some potential data sources were unavailable to us. The global focus of the study is broad, as many online forums are not country or location-specific, and there were insufficient online transgender forums available to us to focus specifically on the Australian context. As with any online research, we are unable to correctly identify if the person who posted in the forums we examined were telling the truth of who they actually were. Although the internet can be a rich source of research data, deception can be easily done in online communication [35]. However, we did carefully scrutinize the stories they posted, there was commonality across accounts in the data we presented in the paper, which were also cross-checked by a trans woman for plausibility.

## 5. Conclusions

Trans women participating in online forums in our study face many challenges in being a transgender person. In embodying their transgender, many interact with others in society as well as health care providers. It is crucial that we understand the ways trans women to express their gender identity. Trans women have heterogeneous experiences which often do not match those of expected discourses (and diagnoses) which have implications for access to treatments to achieve their desired gender identity. We argue that transgender individuals have the rights to carry out self-determination practice as a way to achieve their gender autonomy. Health care providers must understand the way trans women embody their identity as one of the myriad diverse human expressions of one’s own gender.
